# O-Antigen Delays Lipopolysaccharide Recognition and Impairs Antibacterial Host Defense in Murine Intestinal Epithelial Cells

**DOI:** 10.1371/journal.ppat.1000567

**Published:** 2009-09-04

**Authors:** Claudia U. Duerr, Sebastian F. Zenk, Cécilia Chassin, Johanna Pott, Dominique Gütle, Michael Hensel, Mathias W. Hornef

**Affiliations:** 1 Institute for Medical Microbiology and Hospital Epidemiology, Hannover Medical School, Hannover, Germany; 2 Institute for Microbiology, University Hospital Erlangen, Erlangen, Germany; 3 Division of Infection Biology, University Hospital Erlangen, Erlangen, Germany; University of Toronto, Canada

## Abstract

Although Toll-like receptor (TLR) 4 signals from the cell surface of myeloid cells, it is restricted to an intracellular compartment and requires ligand internalization in intestinal epithelial cells (IECs). Yet, the functional consequence of cell-type specific receptor localization and uptake-dependent lipopolysaccharide (LPS) recognition is unknown. Here, we demonstrate a strikingly delayed activation of IECs but not macrophages by wildtype *Salmonella enterica* subsp. *enterica* sv. (*S.*) Typhimurium as compared to isogenic O-antigen deficient mutants. Delayed epithelial activation is associated with impaired LPS internalization and retarded TLR4-mediated immune recognition. The O-antigen-mediated evasion from early epithelial innate immune activation significantly enhances intraepithelial bacterial survival *in vitro* and *in vivo* following oral challenge. These data identify O-antigen expression as an innate immune evasion mechanism during apical intestinal epithelial invasion and illustrate the importance of early innate immune recognition for efficient host defense against invading *Salmonella*.

## Author Summary

The mammalian host recognizes infection by the detection of particular microbial structures. Recognition of these structures leads to activation of host defense effector mechanisms that in turn combat infection. A very potent activating microbial structure is lipopolysaccharide, a cell wall component released by many bacteria such as *Salmonella*, one of the most frequent causative agents of foodborne infection of the gut. We previously showed that cells lining the gut surface require uptake of bacterial lipopolysaccharide for its detection. The functional consequence of lipopolysaccharide uptake, however, was unknown. Here, we demonstrate that the uptake of lipopolysaccharide released by *Salmonella* is impaired by its extensive sugar modification. Impaired lipopolysaccharide uptake prevents early activation of host defense mechanisms and thereby allows *Salmonella* to better survive and proliferate within the host's intestinal cells. Thus, this lipopolysaccharide modification represents a mechanism by which *Salmonella* impairs recognition by the mammalian host to more efficiently cause infection of the intestinal mucosa.

## Introduction

Lipopolysaccharide (LPS) is an obligate constituent of the outer membrane of gram-negative bacteria. It is composed of three parts - a conserved lipid A, a short core carbohydrate, and the O-antigen assembled by a variable number of highly polymorphic carbohydrate subunits [1]. The lipid A consists of a hexa-acylated disaccharide. It is the ligand of the innate immune receptor Toll-like receptor (TLR) 4 and represents one of the most potent immunostimulatory molecules. TLR4-mediated LPS recognition provides an important signal for activation of the antimicrobial host defense during bacterial infection [2],[3]. The O-antigen confers resistance to serum complement activation during systemic infection and represents the chemical basis of bacterial serotyping [4].

Due to its amphiphilic character, LPS forms aggregates in watery solution. The serum protein LPS-binding protein (LBP) retrieves LPS from aggregates or intact bacteria and transfers it to the GPI-anchored surface-bound or soluble form of CD14. CD14 in turn presents the LPS molecule to the MD-2/TLR4 receptor complex. Alternatively, LPS bound to soluble MD-2 can bind to the TLR4 receptor facilitating efficient recognition of even minute amounts of LPS [5],[6]. The structural basis of this intense interaction has recently been resolved [7]. Ligand binding induces a conformational change of the TLR4 dimer and leads to signal transduction, transcriptional activation, and the production and secretion of proinflammatory mediators. Beside professional immune cells, also other cell types such as epithelial cells express functionally active innate immune receptors [8],[9]. Lack of TLR4 signaling has been associated with enhanced susceptibility to microbial challenge, increased tissue destruction during mucosal injury and cancerogenesis within the intestinal tract [10],[11],[12].

Strikingly, the subcellular localization of TLR4 has been demonstrated to differ between macrophages and intestinal epithelial cells (IEC) [13]. Myeloid cells harbor TLR4 on the cell surface and ligand recognition and signling occur from the plasma membrane [14]. In contrast TLR4 is restricted to an intracellular compartment in IECs [13],[15]. LPS is rapidly internalized, reaches the TLR4-positive compartment and initiates signal transduction [16]. Although LPS internalization has been noted since many years [13],[14],[17],[18] and the intracellular TLR4 localization has been confirmed in pulmonary, renal and corneal epithelial cells as well as endothelial cells [15], [17], [19]–[22], the functional consequence of the different cellular localization of the TLR4 molecule and the functional role of ligand internalization is unknown.

Here we report a strikingly delayed recognition of wildtype *Salmonella* as compared to O-antigen deficient *Salmonella* by IECs but not macrophages. Delayed recognition of wildtype *Salmonella* is caused by lack of early TLR4-mediated cell activation associated with impaired LPS internalization. Importantly, lack of early epithelial activation significantly promotes intraepithelial bacterial survival and O-antigen expression is linked to enhanced numbers of intraepithelial *Salmonella* after oral infection *in vivo*. The data show that O-antigen expression contributes to bacterial virulence during apical epithelial invasion prior to contact with serum complement and illustrate the susceptibility of *Salmonella* to antibacterial defense activation before it reaches and establishes its protected intracellular niche.

## Results

### Early activation of IECs after exposure to O-antigen deficient *Salmonella*

In order to evaluate a possible biological effect of LPS glycosylation on epithelial cell stimulation, differentiated and polarized intestinal epithelial m-IC_cl2_ cells were coincubated with wildtype *Salmonella*, isogenic O-antigen deficient mutants, or their respective complemented strains. The *waaG* (*rfaG*) gene encodes a UDP-glucose:(heptosyl)LPS α1,3-glucosyltransferase and mutants exhibit a rough Rd_1_ LPS phenotype with only the inner core sugars attached to the lipid A molecule [23],[24]. *waaL* (*rfaL*) encodes the O-antigen ligase, the last step in the LPS biosynthesis. WaaL functions within the periplasmic space at the cytoplasmic membrane to ligate the presynthesized O-antigen chain onto the lipid A core molecule [1],[24]. *waaL* mutants therefore express the complete core sugars but completely lack the O-antigen (Ra LPS). O-antigen expression was confirmed using silver staining of LPS extracts (Fig. S1A).

**Figure 1:**
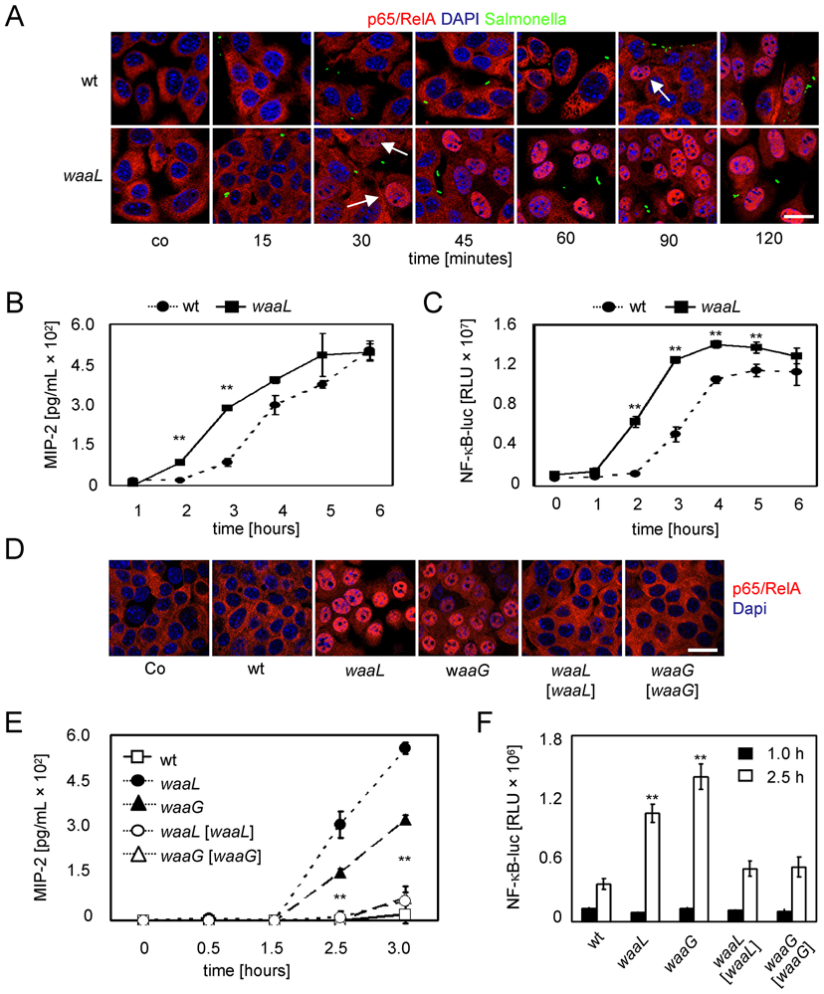
Expression of O-antigen confers delayed epithelial activation. (A) m-IC_cl2_ cells were exposed to wildtype *S.* Typhimurium and to isogenic O-antigen deficient *waaL* mutants, both carrying a constitutive GFP plasmid. Nuclear translocation of the NF-κB subunit p65/RelA was visualized by immunostaining. The arrows mark the earliest detectable p65/RelA translocation. Bar, 5 µm. (B) m-IC_cl2_ cells or (C) m-IC_cl2_ cells stably transfected with a NF-κB-luciferase construct were exposed to *S.* Typhimurium wildtype, or an isogenic O-antigen deficient *waaL* mutant, and the secretion of MIP-2, or luciferase synthesis, respectively, was quantified after the indicated time. (D) m-IC_cl2_ cells were exposed to wildtype *S.* Typhimurium, two isogenic O-antigen deficient mutants (*waaL* and *waaG*), as well as their respective complemented controls for 1 h and the nuclear translocation of the NF-κB subunit p65/RelA was visualized by immunostaining. Bar, 5 µm. (E) m-IC_cl2_ cells, or (F) m-IC_cl2_ cells stably transfected with a NF-κB-luciferase construct were exposed to wildtype *S.* Typhimurium, the isogenic O-antigen deficient *waaL* and *waaG* mutants, and their respective complemented control strains, and secretion of MIP-2, or luciferase synthesis, respectively, was quantified after the indicated time points. A multiplicity of infection (MOI) of 10 was chosen for all experiments. All data presented are representative for at least three independent experiments. The asterisks indicate a significant difference between the respective rough LPS mutant (Δ*waaL* or Δ*waaG*) as compared to all wildtype and complemented *Salmonella* strains; **, p<0.01.

Cellular activation was evaluated using (i) visualization of nuclear translocation of the NF-κB subunit p65/RelA, (ii) a stably transfected transcriptional NF-κB luciferase reporter construct, and (iii) quantification of the secreted proinflammatory chemokine MIP-2. Strikingly, a significant difference in the kinetics of cellular activation was recognized after challenge with wildtype and LPS mutant strains. Whereas no difference in the overall magnitude of epithelial cell activation was noted, *waaL* mutants induced a significantly earlier p65/RelA translocation (Fig. 1A, the earliest detectable p65/RelA translocation is marked with arrows) and an accelerated course of chemokine secretion and NF-κB reporter gene transcription in epithelial cells as compared to wildtype *Salmonella* (Fig. 1B and C). This difference in p65/RelA translocation (Fig. 1D), chemokine secretion (Fig. 1E), and NF-κB reporter gene activation (Fig. 1F) was similarly observed using *waaG-* and *waaL*-deficient mutants and reversed by the complemented strains carrying an expression plasmid encoding the *waaG* and *waaL* gene, respectively. Thus lack of O-antigen expression leads to a significantly accelerated recognition of *Salmonella* by IECs.

### TLR4 significantly contributes to innate immune recognition of *Salmonella* by intestinal epithelial cells

**Figure 2:**
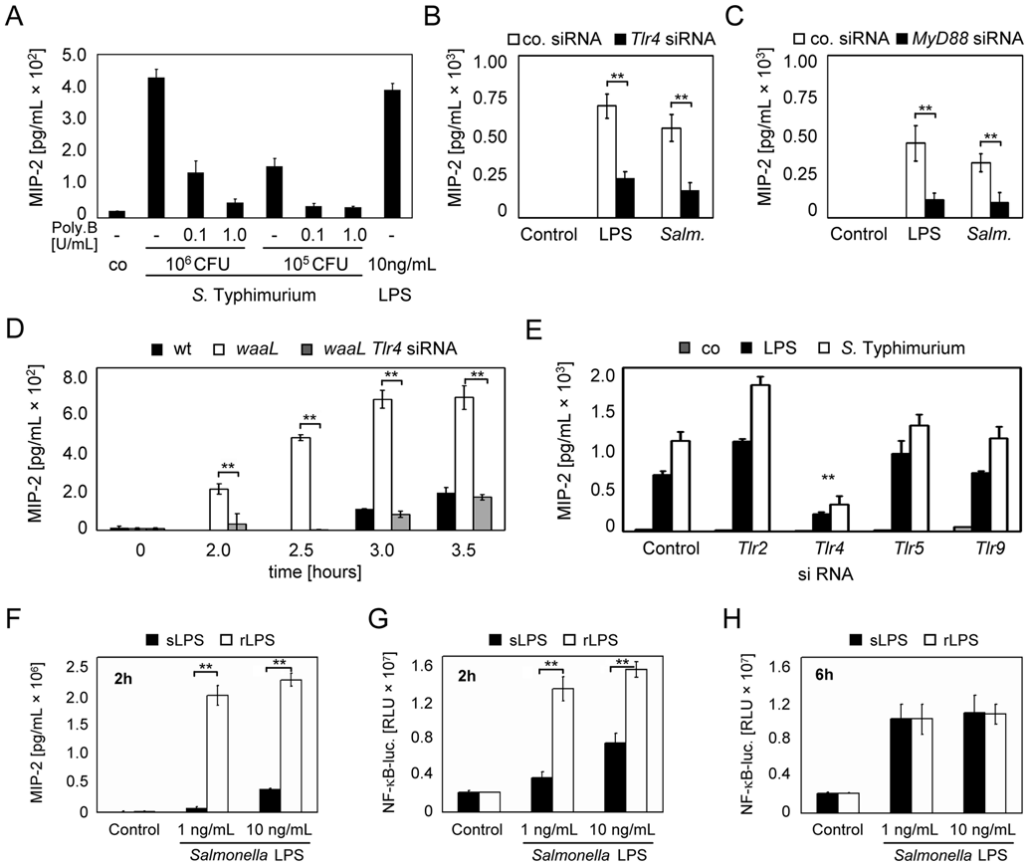
LPS recognition is critical for early epithelial activation by *Salmonella*. (A) Wildtype *S.* Typhimurium were coincubated with confluent m-IC_cl2_ cells at a MOI of 10∶1 (10^6^/mL) or 1∶1 (10^5^/mL) for 1 h and the cell culture supernatant was removed, sterile filtered, and used to stimulate naive m-IC_cl2_ cells in the absence, or presence of the indicated concentrations of polymyxin B for 6 h. Rough LPS (D31m4) was included as control. (B and C) MIP-2 secretion by m-IC_cl2_ cells treated with control-, and (B) *Tlr4*-specific, or (C) *MyD88*-specific siRNA 6 h after exposure to rough LPS (10 ng/mL) or wildtype *S.* Typhimurium at a MOI of 10∶1. (D) MIP-2 secretion by m-IC_cl2_ cells exposed to wildtype *Salmonella*, or an isogenic *waaL*-mutant for the indicated time after treatment with control, or *Tlr4*-specific siRNA. (E) MIP-2 secretion by m-IC_cl2_ cells treated with control, *Tlr2-*, *Tlr4-*, *Tlr5*, or *Tlr9*-specific siRNA 6 h after exposure to rough LPS, or wildtype *S.* Typhimurium. (F–H) m-IC_cl2_ cells (F), or stably transfected m-IC_cl2_ cells expressing a NF-κB-luciferase construct (G and H), were exposed to *Salmonella* smooth LPS (sLPS), or *Salmonella* rough LPS (rLPS) at the indicated concentrations for 2 hours (F and G), or 6 hours (H) and the secreted MIP-2, or luciferase, respectively, was quantified. **, p<0.01.

A similar delay in epithelial activation by wildtype *Salmonella* was also noted using heat-killed or UV-treated *Salmonella* suggesting structural impairment of LPS recognition by the O-antigen rather than O-antigen-mediated active inhibition of epithelial cell activation (Fig. S1B and data not shown). To examine the contribution of TLR4-mediated epithelial cell activation and exclude indirect effects of the O-antigen on cellular activation, the role of TLR4 in *Salmonella* recognition during coculture with IECs over 6 hours was examined. First the stimulatory activity released in the cell culture medium at bacterial numbers corresponding to a multiplicity of infection (MOI) of 10∶1 (10^6^ CFU/mL) and 1∶1 (10^5^ CFU/mL) during one hour was completely inhibited by addition of the LPS-inhibiting agent polymyxin B (Fig. 2A). Also, inhibition of *Tlr4* (Fig. 2B) or *Myd88* (Fig. 2C) expression by small interfering (si) RNA technique inhibited epithelial activation by *Salmonella* to a similar degree as epithelial activation by LPS. In fact, early recognition of the O-antigen deficient *waaL* mutant *Salmonella* was almost abolished in epithelial cells treated with *Tlr4* siRNA (Fig. 2D). In contrast, inhibition of TLR2, TLR5, or TLR9 expression did not reduce the epithelial response to bacterial exposure (Fig. 2E). Consistently, no early epithelial stimulation was observed after apical exposure to other innate immune receptor ligands released by gram-negative bacteria such as flagellin, di- or tri-acylated lipopeptides, or CpG oligonucleotides (data not shown). The important role of LPS for the observed effect of delayed recognition of wildtype *Salmonella* was finally confirmed using LPS purified from O-antigen positive (smooth-type LPS, sLPS) as well as O-antigen negative (rough-type LPS, rLPS) *Salmonella*. Indeed, a similar pattern as compared to exposure to whole wildtype and mutant *Salmonella* with delayed epithelial activation in response to smooth LPS at early time points (Fig. 2F and G) but similar levels of epithelial activation at later time points (Fig. 2H) was observed. Thus epithelial activation early during the time course of coculture is predominantly caused by TLR4-mediated cell stimulation. The observed delay in the recognition of smooth *Salmonella* is not related to an O-antigen-mediated suppressive effect on early epithelial activation but rather caused by an inhibitory effect of the O-antigen on LPS recognition by epithelial TLR4.

### IECs but not macrophages show delayed immune activation by wildtype *Salmonella*

**Figure 3:**
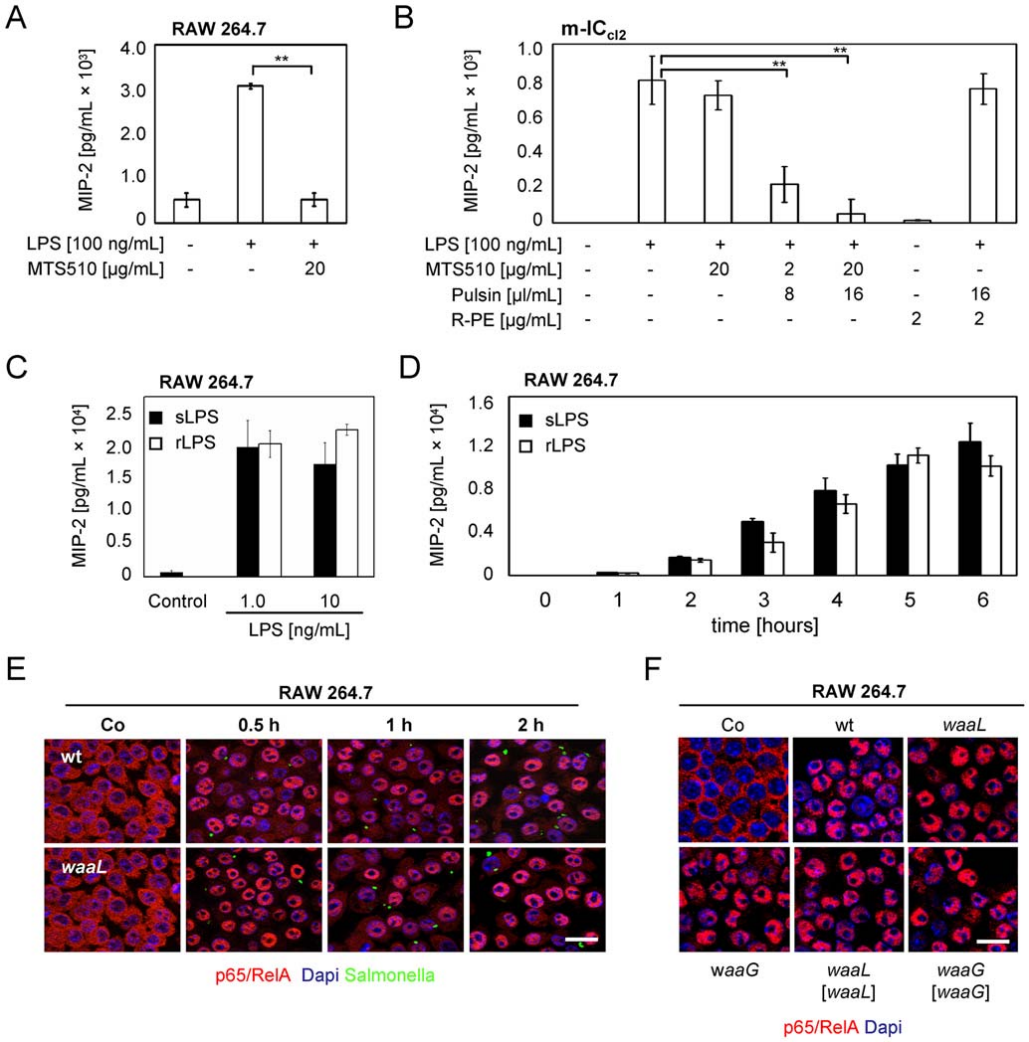
Delayed recognition is not observed in macrophages carrying TLR4 on the plasma membrane. (A) Anti-TLR4/MD2 antibody treatment abolishes LPS-mediated stimulation of RAW 264.7 macrophages. (B) In contrast, anti-TLR4/MD2 antibody-mediated inhibition of LPS-induced m-IC_cl2_ cell activation requires the presence of the protein delivery reagent PULSin. (C and D) RAW 264.7 macrophages were exposed to smooth LPS (sLPS), or rough LPS (rLPS), at the indicated concentrations for 2 hours (C), or at 10 ng/mL for the indicated time points (D), and the amount of secreted MIP-2 was quantified. (E and F) RAW 264.7 macrophages were coincubated with wildtype *S.* Typhimurium or an isogenic O-antigen deficient *waaL* mutant carrying a constitutive GFP plasmid for the indicated time points, and nuclear translocation of the NF-κB subunit p65/RelA was visualized by immunostaining. (F) RAW 264.7 macrophages were coincubated with wildtype *S.* Typhimurium, O-antigen deficient Δ*waaL* or Δ*waaG* mutants, or their respective complemented strains for one hour and nuclear translocation of the NF-κB subunit p65/RelA was visualized by immunostaining. Bar, 10 µm. **, p<0.01.

Myeloid cells like macrophages carry the TLR4 receptor complex on the cell surface and signaling is initiated at the plasma membrane [14]. This is in contrast to IEC lines and isolated primary IECs that exhibit restriction of the TLR4 molecule to an intracellular compartment [13],[15]. In these cells, receptor activation requires ligand internalization and signaling is initiated at the intracellular TLR4-positive compartment [17]. Using the protein delivery reagent PULSin in combination with TLR4/MD2 blocking antibodies, the different receptor localization could be functionally demonstrated. Whereas activation of myeloid cells was readily blocked by addition of the blocking anti-TLR4 antibody MTS510 to the cell culture medium, antibody-mediated inhibition of epithelial activation was only observed in the presence of the protein delivery reagent PULSin (Fig. 3A and B). Interestingly, both, sLPS - as well as rLPS - stimulated RAW 264.7 cells at early time points to a similar degree and with very similar kinetics (Fig. 3C and D). Also, early p65/RelA nuclear translocation was similarly induced in macrophages by all strains, wildtype as well as Δ*waaL* and Δ*waaG* mutant *Salmonella* as well as the respective complemented strains (Fig. 3E and F). Thus the delayed recognition of wildtype sLPS as compared to rLPS is restricted to IECs that are devoid of plasma membrane expression of TLR4 and rely on ligand internalization. Yet we cannot exclude that macrophage activation additionally occurs by LPS release during phagocytosis.

### The kinetics of *Salmonella* recognition is not influenced by bacterial invasion

**Figure 4:**
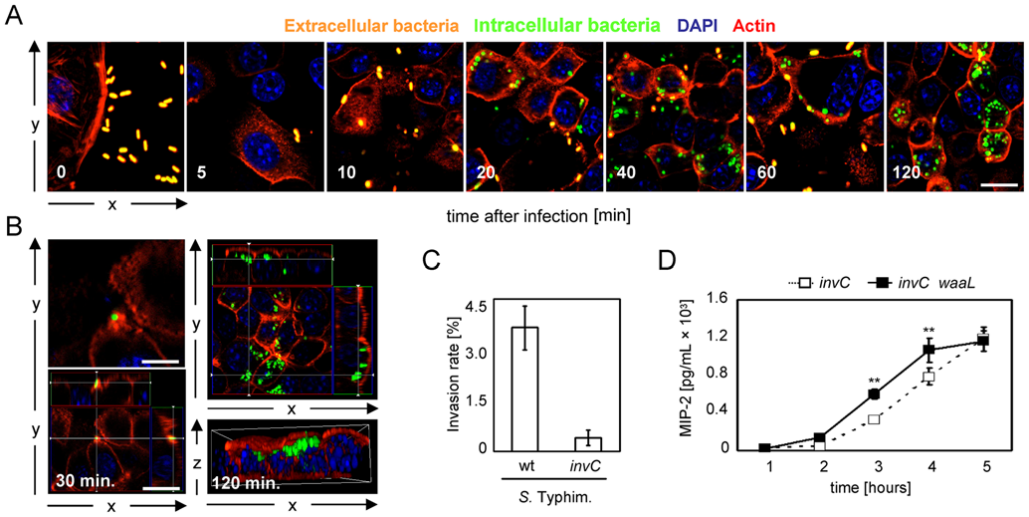
Delayed activation by wildtype *Salmonella* is independent of bacterial invasion. (A) m-IC_cl2_ cells were coincubated with constitutively GFP-expressing wildtype *S.* Typhimurium for the indicated time points, fixed, and visualized by immunostaining using a mixture of two mouse monoclonal anti-*Salmonella* O-antigen (anti-O4 and anti-O5) antibodies in combination with a Texas-red-conjugated secondary antibody (red) in the absence of cell permeabilization. This facilitates the differentiation of intracellular bacteria (green) and extracellular bacteria (green plus red = orange). Counterstaining was performed using TR-phalloidin (red), and Dapi (blue), to reveal the cytoskeleton and the nuclei, respectively. Scale bar, 5 µm. (B) The invasive behaviour (left panel), and intracellular presence (right panel) of wildtype *S.* Typhimurium in m-IC_cl2_ cells is illustrated 30 min and 2 hours after infection. The lower right image shows a three-dimensional reconstruction of the polarized epithelial cell layer from a lateral view. Bar, 5 µm. (C) The non-invasive phenotype of a *S.* Typhimurium *invC* mutant was verified on m-IC_cl2_ cells using a Gentamicin protection-assay. (D) Kinetics of MIP-2 secretion by m-IC_cl2_ cells exposed to O-antigen positive non-invasive *invC* mutant *Salmonella* as compared to O-antigen negative non-invasive *invC waaL* double mutant *Salmonella* [MOI 10∶1]. **, p<0.01.

Genes encoded by the so called *Salmonella* pathogenicity island 1 (SPI-1) confer the ability to invade epithelial cells. Bacterial invasion is induced by direct translocation of effector proteins into the host cell cytoplasm which causes actin polymerization and membrane protrusions. Within one hour, this mechanism leads to bacterial internalization and localization within an endosomal compartment named *Salmonella* containing vacuole (SCV). Initially, the kinetics of *Salmonella* invasion was examined using constitutive GFP-positive wildtype *Salmonella* followed by immunostaining with anti-O-antigen *Salmonella* O4/O5 antibodies without prior cell membrane permeabilization. This technique allows the differentiation of extracellular (simultaneously green and red = orange) and intracellular (green) *Salmonella*. Exposure of confluent polarized m-IC_cl2_ cells revealed bacterial invasion starting approximately 20 minutes after challenge with significant numbers of intracellular bacteria at 2 hours after infection (Fig. 4A). Fig. 4B provides a more detailed illustration of the actin-dependent mode of *Salmonella* invasion at 30 minutes after infection (left panel) and the intracellular localization after 2 hours (right panel). Importantly, the O-antigen-mediated delay in LPS recognition was also observed in invasion-mutants: An isogenic pair of smooth and rough *invC*-mutants exhibited a similar difference in the kinetics of epithelial stimulation as compared to invasion-competent *Salmonella* (Fig. 4C and D). Also, *hilA*- and *pho-24* (PhoP^c^) deficient smooth *Salmonella*, both significantly impaired in epithelial invasion, exhibited a similar pattern of reduced activation at early time points but cellular stimulation at later time points after infection (Fig. S2A and B). Thus the observed delay in epithelial activation by wildtype bacteria is not dependent on their ability to exhibit an epithelial cell-invasive phenotype but rather result from extracellular ligand exposure.

### Delayed recognition of smooth LPS is associated with retarded ligand internalization

**Figure 5:**
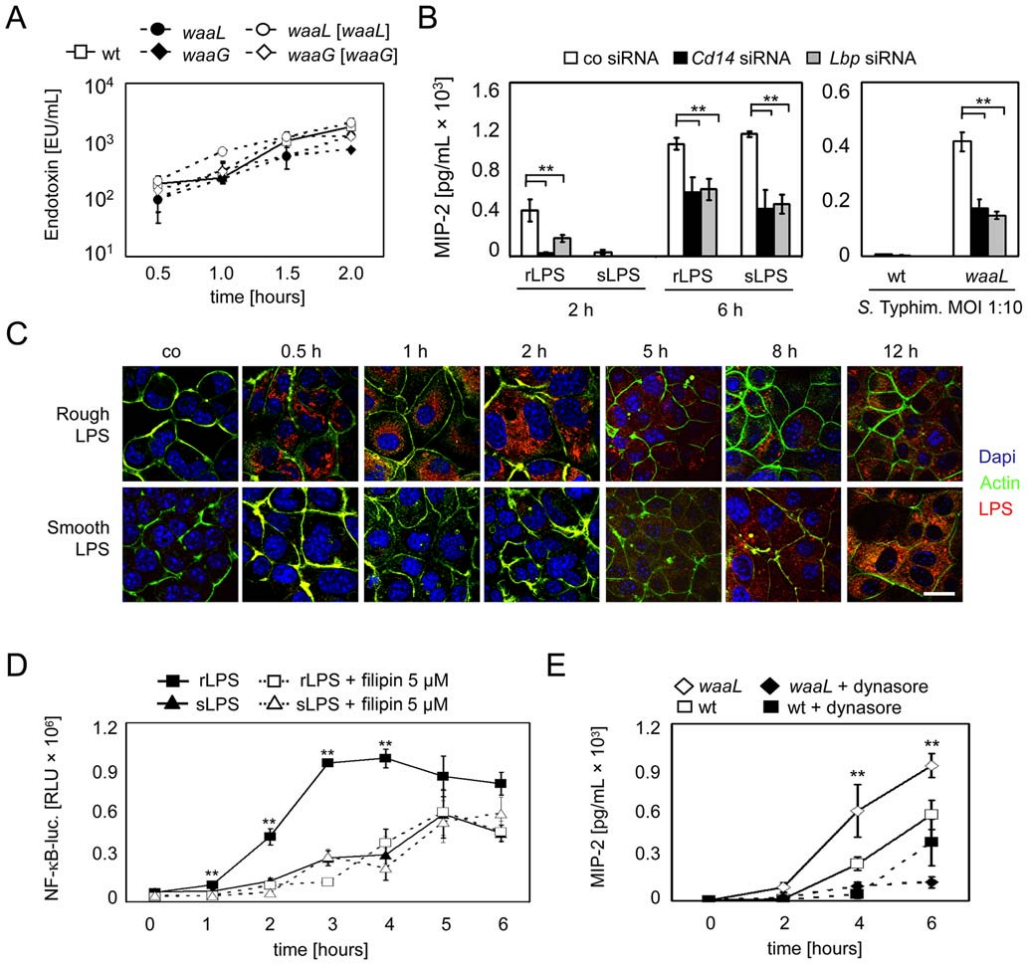
Delayed recognition of wildtype LPS is associated with reduced internalization. (A) Endotoxin released in the cell culture medium from wildtype *S.* Typhimurium, two isogenic O-antigen deficient mutants (*waaL* and *waaG*), as well as their respective complemented controls at a concentration of 10^6^ CFU/mL was quantified using a Limulus assay. (B) MIP-2 secretion by m-IC_cl2_ cells treated with control-, *Cd14*-specific, or *Lbp*-specific siRNA 2 and 6 h after exposure to rough or smooth LPS (10 ng/mL), or 2 h after infection with wildtype and *waaL-*deficient *S.* Typhimurium at a MOI of 10∶1. (C) m-IC_cl2_ cells were exposed to biotin-conjugated rough or smooth LPS for the indicated time points and the internalized LPS was visualized by immunostaining. Counterstaining was performed with MFP488-phalloidin and Dapi. Bar, 5 µm. (D) Stably transfected m-IC_cl2_ cells expressing a NF-κB-luciferase construct were stimulated with rough (rLPS), or smooth (sLPS) in the absence, or presence of filipin for the indicated time points and the amount of luciferase was quantified. (E) MIP-2 secretion by m-IC_cl2_ cells exposed to wildtype or *waaL*-deficient *S.* Typhimurium in the presence or absence of the dynamin inhibitor dynasore. **, p<0.01.

Viable bacteria continuously release LPS in the surrounding medium. In accordance, significant amounts of 10^2^ EU/mL (approximately 10 ng/mL) LPS were found to be released from viable wildtype *Salmonella* into the cell culture medium within 30 minutes. The concentration increased up to 10^3^ EU/mL (approximately 100 ng/mL) during the observed time period of 2 hours. No significant difference in the degree of endotoxin release between wildtype and *waaL-* and *waaG*-mutants or their respective complemented *Salmonella* strains was noted (Fig. 5A). As expected, inhibition of CD14 and LBP expression by siRNA significantly impaired LPS and *Salmonella*-mediated activation of m-IC_cl2_ cells in accordance with the literature (Fig 5B) [25]. Strikingly, LPS internalization studies using biotinylated rLPS or sLPS preparations revealed a marked difference in the kinetics of ligand uptake. Whereas detectable amounts of rLPS were observed after 30 minutes, wildtype LPS remained undetectable until many hours after exposure (Fig 5C). Previous characterization of intestinal epithelial stimulation with rLPS identified a clathrin- and lipid raft-dependent pathway of LPS internalization and receptor activation [15]. Inhibition of lipid raft formation with filipin previously linked to recognition of rLPS abolished early recognition of rLPS but left the more delayed cellular activation induced by wildtype sLPS unaffected (Fig. 5D). Also, a significant inhibitory effect of clathrin siRNA on early recognition of rLPS was noted (data not shown) and dynamin inhibition by dynasore significantly reduced activation by rough, Δ*waaL Salmonella* consistent with this rapid internalization pathway for rLPS uptake (Fig. 5E). These data suggest that qualitative differences in the uptake and intracellular transport mechanism between sLPS and rLPS exist and account for the observed delay in the epithelial recognition of wildtype, O-antigen-positive *Salmonella*. Similar results obtained using viable invasive and non-invasive *Salmonella*, heat-killed *Salmonella*, or purified LPS suggest involvement of plasma membrane-to-Golgi traffic. Yet we cannot exclude that transport pathways from the SCV to the Golgi apparatus are also affected.

### Early innate immune activation restricts the number of intracellular bacteria *in vitro* and *in vivo*

**Figure 6:**
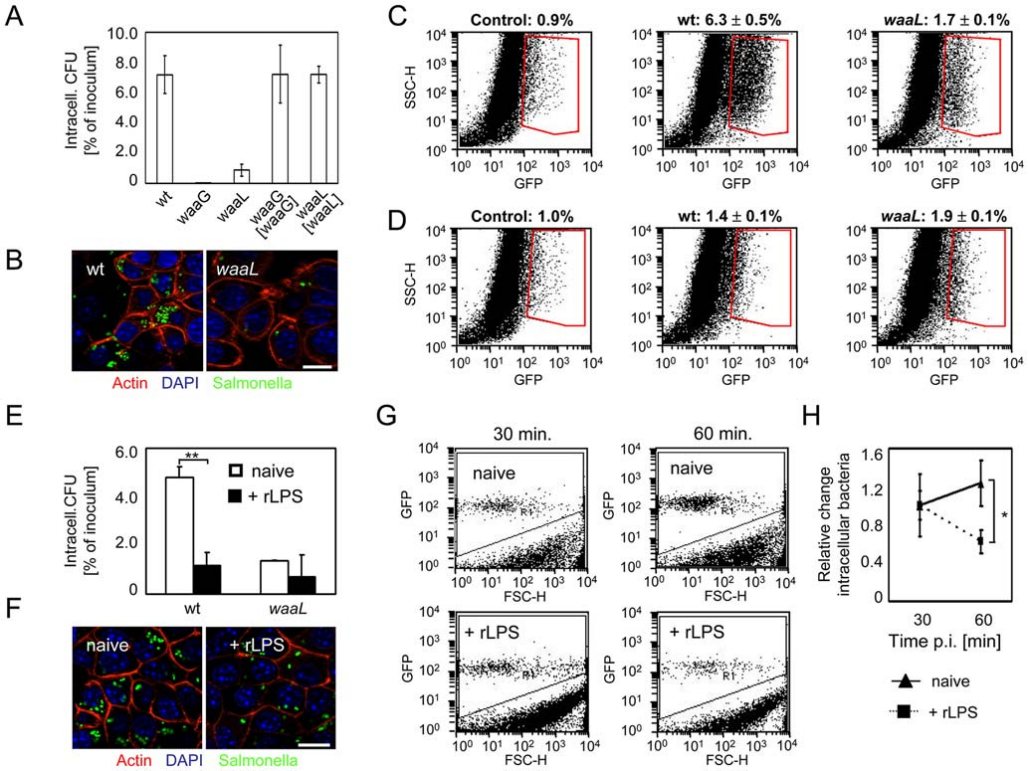
Early epithelial activation affects the number of intracellular bacteria. (A) Number of viable intracellular bacteria 2 hours after infection of m-IC_cl2_ cells with wildtype *S.* Typhimurium, two isogenic O-antigen-deficient mutants (*waaL* and *waaG*), as well as the respective complemented controls. CFU, colony forming units. (B) Immunostaining using GFP-expressing bacteria illustrating the number of intracellular wildtype bacteria and *waaL* mutants 2 hours after infection. Bar, 5 µm. (C and D) Flow cytometric analysis of m-IC_cl2_ cells 2 hours (C) and 30 min (D) after infection with wildtype or *waaL*-deficient *Salmonella*. (E) Viable intracellular wildtype or *waaL*-deficient bacteria 2 hours after infection of naive m-IC_cl2_ cells or cells stimulated with 100 ng/mL rLPS prior to infection. (F) Immunofluorescence staining using GFP-expressing wildtype *Salmonella* illustrating the number of intracellular bacteria at 2 hours after infection of naive or rLPS-stimulated m-IC_cl2_ cells. Bar, 5 µm. (G) Flow cytometric quantification of GFP-expressing wildtype *Salmonella* in a defined volume of epithelial cell lysate 30 and 60 min after infection of naive or rLPS-stimulated m-IC_cl2_ cells (100 ng/mL). (H) The corresponding values depicted as relative change of the number of intracellular wildtype *Salmonella* between 30 and 60 min after infection of naïve or rLPS-stimulated epithelial cells.

To examine the functional consequences of early epithelial activation, a standard Gentamicin protection-assay was performed. Strikingly, both O-antigen negative mutants but not their respective complemented strains exhibited a significantly reduced number of viably intracellular bacteria two hours after infection (Fig. 6A). This was confirmed by immunofluorescence (Fig. 6B) as well as by flow cytometry (Fig. 6C) using GFP expressing wildtype and *waaL-*deficient *Salmonella*. Both, the relative number of *Salmonella*-positive epithelial cells (6.3±0.5% *versus* 1.7±0.1%, p<0.01) as well as the mean fluorescence intensity (MFI) of positive cells indicating the number of bacteria per cell (MFI 487.8±14.1 *versus* MFI 343.9±7.4, p<0.01) were significantly enhanced 2 hours after infection with wildtype as compared to *waaL-*deficient *Salmonella* (Fig. 6C). Notably, this difference in the number of viable *Salmonella* was not due to impaired invasion of *waaL-*deficient *Salmonella* since similar numbers of intracellular bacteria were obtained 30 minutes after infection (1.4±0.1% *versus* 1.9±0.1%) (Fig. 6D). In fact flow cytometric quantification of intracellular bacteria after epithelial cell lysis revealed an approximately 2-fold enhanced invasion rate of the O-antigen deficient *waaL* mutant *Salmonella* as compared to wildtype bacteria (Fig. S1C and D). The increase of the number and fluorescence intensity of wildtype *Salmonella*-infected epithelial cells together with the marked clusters of intracellular wildtype *Salmonella* 2 hours after infection suggest significant intraepithelial proliferation early after invasion. In contrast, no signs of bacterial growth were noted for the *waaL*-deficient *Salmonella* strain. In addition, reduced bacterial numbers in epithelial cells did not appear to result from general growth or viability defects of O-antigen deficient *Salmonella*. Wildtype, Δ*waaL*, and Δ*waaG Salmonella* as well as the complemented mutants exhibited comparable growth rates in LB medium, or m-IC_cl2_ cell lysate (Fig. S1E). Also, both wildtype and Δ*waaL* or Δ*waaG Salmonella* were able to induce persistent intracellular infection in m-IC_cl2_ cells (Fig. S1F). In accordance with the different phenotype observed in epithelial cells and macrophages (Fig. 1 *versus* Fig. 3), the intracellular survival illustrated by enhanced fluorescence of infected cells with wildtype *Salmonella* was only found in epithelial cells. In contrast, the fluorescence of *Salmonella*-infected RAW 264.7 macrophages was not significantly altered during the first 2 hours of infection, irrespective of the *Salmonella* strain used (Fig. S2C). Of note, an enhanced internalization of the *waaL*-deficient *Salmonella* mutant was observed after macrophage infection in accordance with Ilg *et al.* [26] (Fig. S2C).

To confirm that the observed antibacterial effect was directly linked to early innate immune recognition and cell activation, epithelial cells were infected with wildtype bacteria in the presence or absence of rLPS. Indeed, the number of intracellular bacteria as measured by invasion assay (Fig. 6E), or immunofluorescence (Fig. 6F) was significantly reduced in rLPS-stimulated epithelial cells illustrating the critical importance of early cell activation to restrict intracellular bacterial growth. Wildtype *Salmonella* in sLPS-stimulated epithelial cells were significantly less affected (Fig. S2D). The dramatic nature of this antibacterial effect was illustrated by flow cytometric quantification of intracellular bacteria in cell lysate between 30 and 60 minutes after infection. Whereas invasion of naïve epithelial cells allowed immediate intracellular bacterial growth, rLPS-stimulated epithelial cells were able to restrict the number of *Salmonella* (Fig. 6G and H). Thus early activation of intestinal epithelial cells by O-antigen-deficient *Salmonella* is associated with significantly reduced intraepithelial survival.

**Figure 7:**
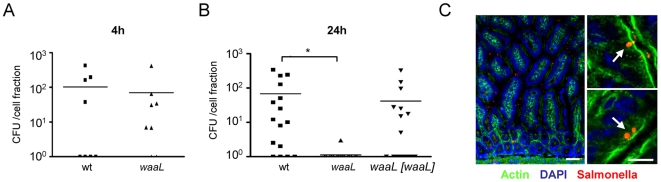
Early epithelial stimulation restricts the number of intraepithelial *Salmonella in vivo*. Colony forming units (CFU) cultured from isolated highly pure (>98% E-cadherin^+^/CD45^−^) intestinal epithelial cells (IEC) (A) 4 h (n = 8 mice per group) and (B) 24 h (n = 16 mice per group) after oral infection of Balb/c mice with 1×10^8^ CFU wildtype (wt), *waaL*-deficent (*waaL*), and complemented *waaL* [*waaL*] Salmonella. *, p<0.05. (C) Immunostaining for *S.* Typhimurium O-antigen (anti-O4 and anti-O5) in intestinal tissue sections obtained from orally infected mice. Magnification, Bar 50 µm (left panel) and 5 µm (right panel).

*Salmonella* has been shown to invade IECs *in vivo* after oral challenge [27]. Intestinal epithelial invasion from the luminal side occurs without prior contact with tissue macrophages or complement. To examine a possible effect of O-antigen expression on intraepithelial survival *in vivo*, mice were orally challenged and highly pure IECs were isolated and examined for the presence of viable *Salmonella*. Similar numbers of intracellular wildtype and *waaL*-deficient *Salmonella* were noted at early time points following infection (Fig. 7A). Interestingly, a significant reduction of O-antigen-deficient (*waaL*) *Salmonella* as compared to wildtype as well as the respective complemented *Salmonella* was detected in highly pure IECs later during the course of infection (Fig. 7B). The presence of intraepithelial wildtype *Salmonella* after oral challenge was also confirmed by immunohistology (Fig. 7C). Thus, lack of O-antigen expression does not influence intestinal epithelial invasion but intraepithelial survival of *Salmonella in vitro* and *in vivo*. These results identify O-antigen expression as innate immune evasion strategy to enhance intraepithelial survival. O-antigen expression might thereby promote intraepithelial proliferation and mucosal spread.

## Discussion

*S.* Typhimurium is one of the leading causative agents of enteritis in humans. Infection is acquired by oral ingestion of contaminated food. In the intestine, *Salmonella* firmly attaches to the epithelial surface and induces membrane protrusions that surround the bacterium and form an endosomal vesicle called *Salmonella*-containing vacuole (SCV). This process has been extensively studied *in vitro* but also confirmed *in vivo* [27],[28]. Intestinal epithelial invasion from the enteric lumen occurs prior to contact with serum complement or professional phagocytes such as macrophages. It plays an important role in the induction of enteritis and mucosal damage *in vivo* and thus represents an essential step in *Salmonella* pathogenesis [29],[30].

Similar to professional immune cells, also intestinal epithelial cells express receptors of the innate immune system, and thus might contribute to recognition of microbial infection and antibacterial host defense during the initial phase of infection. Indeed, the LPS structure was shown to significantly influence epithelial invasion [26],[31]. Also, innate immune recognition via TLR4 was reported to play a significant role in the host defense against *Salmonella* infection *in vivo* [10], [32]–[35]. Strikingly, the subcellular localization of TLR4 in myeloid versus epithelial cells is markedly different. Whereas the receptor molecule is situated on the cell surface of macrophages and ligand recognition and cell signaling occurs at the cell membrane, TLR4 in IECs is restricted to the intracellular compartment and ligand recognition requires uptake and intact cell traffic [13],[15],[16]. We could previously show that internalization of rLPS results in significant intracellular accumulation within minutes after exposure [16]. In the present study we show that qualitative differences in the uptake and intracellular transport mechanism between rLPS and sLPS might significantly contribute to immune evasion of wildtype *Salmonella* during the early phase of mucosal infection. Thus our results for the first time report on a biological consequence of intracellular TLR4 localization in IECs. Since apical invasion of enterocytes by *Salmonella* occurs prior to contact with serum complement or professional phagocytes such as tissue macrophages, the epithelial specific delay in wildtype *Salmonella* LPS recognition significantly contributes to bacterial virulence at the mucosal surface in addition to what has been described as serum resistance during systemic spread of the bacteria (Fig. S2E).

LPS is composed of the hydrophobic lipid A, the core polysaccharides, and the highly polymorphic and hydrophilic immunodominant O-antigen [1]. The LPS receptor TLR4 specifically interacts with and recognizes the lipid A part of the LPS molecule. Therefore, even small variations observed in the lipid A structure and their influence on TLR4-mediated recognition have extensively been studied [36]. The O-antigen is not required for the immunostimulatory activity of LPS and variations of the O-antigen and their impact on TLR4-mediated recognition have only recently attracted attention [37],[38]. The O-antigen is composed of up to 100 repetitive structurally variable carbohydrate subunits and the distinction of different O-antigen subunits has been used in the serotyping of various gram-negative bacteria. It is synthesized separately from the rest of the LPS molecule on a lipid carrier by enzymes encoded by the *rfb*/*waa* locus. The O-antigen chain is subsequently transferred to the periplasmic space where ligation to the lipid A-core polysaccharide precursor takes place. Only then, the completed LPS molecule is transferred to the bacterial cell surface [39]. The O-antigen is the major determinant of complement resistance and thus represents an important virulence factor [40]. Indeed, gram-negative enteropathogenic bacteria isolated from fecal samples of diseased patients such as *Yersinia enterocolitica*, *Salmonella enterica*, *Shigella dysenteriae*, as well as enterohemorrhagic (EHEC) or enteropathogenic (EPEC) *Escherichia coli* exhibit long O-antigen chains on their respective LPS molecule. Modifications within the lipid A portion of the molecule have been described to alter the stimulatory potential of LPS [41]. The presence or absence of the O-antigen, however, has not been linked to alterations in the TLR4-mediated signaling cascade leading to MAP kinase and NF-κB activation [38].

Using X-ray diffraction of dried LPS, Kastowsky and collegues estimated the size of the lipid A molecule to measure approximately 2.4 nm in length [42]. Addition of the inner core carbohydrates (corresponding to the LPS produced by the *waaG* mutant *Salmonella*) would result in a length of approximately 3.5 nm, addition of the outer core carbohydrates (corresponding to the LPS produced by the *waaL* mutant *Salmonella*) in a length of approximately 4.4 nm. Their analysis further suggested an additional length of 1.1 to 1.6 nm per repeating carbohydrate unit of the O-antigen. A full length O-antigen with up to 100 repeating units may therefore extend the molecular length to more than 100 nm. The tertiary structure and orientation of long chain O-antigen in respect to the outer cell membrane is not fully understood [42]. Although the O-antigen might be heavily coiled and allow (and actually favor) some degree of lateral bending [42],[43], addition of this long chain hydrophilic residue might dramatically enhance the spacial extension of the LPS molecule [42]–[44]. In accordance, electron microscopic images from the membrane of gram-negative bacteria suggest that the O-antigen extends from the outer cell membrane for 40–100 nm [43],[44]. The length of the extending O-antigen structures are also illustrated by reports on O-antigen mediated impairment of efficient type III secretion in enteropathogenic *Shigella* [45]. Taking into account that the inner diameter of clathrin coated vesicles is strictly defined; one explanation for the delayed internalization of smooth LPS by epithelial cells might therefore be its physical size.

Both *in vitro* as well as *in vivo* experiments revealed comparable intestinal epithelial invasion by wildtype and O-antigen-deficient bacteria at early time points after challenge. Once inside the epithelial cell, *Salmonella* is able to interfere with cellular processes of endosomal maturation altering the molecular composition of its surrounding membranous compartment for its own benefit [46]. Well-established virulence determinants such as the PhoPQ regulon and the SPI-2 type III secretion system contribute to this immune evasive behavior [47]. Avoidance of epithelial activation might significantly contribute to bacterial survival since innate immune signaling has been suggested to promote maturation of endosomal compartments and to influence intracellular bacterial proliferation [48],[49]. Indeed the intracellular viability of O-antigen-deficient *Salmonella* in intestinal epithelial cells *in vitro* and *in vivo* was significantly reduced. Previous animal studies have indicated a significant effect of *Salmonella* O-antigen expression after oral but not intraperitoneal or intravenous infection [50],[51]. Our results provide an explanation for these findings and demonstrate that the O-antigen-modification of LPS significantly contributes to mucosal immune evasion and thus bacterial virulence in the intestine. Our data further point towards a role of intestinal epithelial infection for enteric bacterial multiplication and fecal excretion and thereby transmission of enteropathogenic bacteria like *Salmonella*.

In conclusion, we for the first time provide a functional consequence of internalization-dependent ligand recognition by TLR4 as compared to surface recognition in myeloid cells. We demonstrate that O-antigen modification of *Salmonella* LPS hinders rapid epithelial internalization and delays TLR4-mediated recognition. Evasion from early innate immune activation of IECs markedly enhances intracellular proliferation of wildtype *Salmonella*. This novel immune evasion mechanism might thus significantly contribute to mucosal virulence of enteropathogenic bacteria.

## Materials and Methods

### Ethics statement

Animals were handled in strict accordance with good animal practice as defined by the relevant local animal welfare bodies, and all animal work was approved by the appropriate committee (Landesamt für Lebensmittelsicherheit und Verbraucherschutz, Oldenburg, 07/1334).

### Antibodies and reagents

Antibodies against p65/RelA were purchased from Santa Cruz Biotechnology, Inc. (Santa Cruz, CA). The rat monoclonal anti-TLR4/MD-2 antibody (MTS510) and the mouse monoclonal anti-O4 and anti-O5 antigen antibody were kindly provided by K. Miyaki (Saga Medical School, Nabeshima, Saga, Japan) and M. Kim (Kim Laboratories Inc., Champaign, IL), respectively. All fluorophore-conjugated secondary antibodies and Cy5-conjugated streptavidin were from Jackson ImmunoResearch (West Grove, PA). Filipin, dynasore, and polymyxin B were purchased from Sigma (Taufkirchen, Germany). Plasmid DNA was prepared using the EndoFree Plasmid kit from Qiagen (Hilden, Germany). High purity grade smooth and rough form LPS were purchased from List Biochemicals (Campbell, CA) and Alexis Biochemicals (Lausen, Switzerland) and tested for its TLR4-specific activity using *Tlr4*-deficient C57BL/10ScN-*Tlr4^lps-del^*/JthJ mice (The Jackson Laboratory, Bar Harbor, USA) (Fig. S1G–I). LPS was biotinylated using EZ link biotinylation kit from Thermo Scientific (Rockford, IL). Endotoxin was quantified using the chromogenic QCL-1000 Limulus amebocyte lysate system from Lonza (Basel, Switzerland). All siRNA probes used (*Tlr2*, *Tlr4*, *Tlr5*, *Tlr9*, *Myd88*, *Cd14*, *Lbp*, *Clathrin* and control siRNA) were from Qiagen (Hilden, Germany). For plasmid transfection, siRNA transfection, and intracellular antibody delivery Lipofectamin 2000 (Invitrogen, Carlsbad, CA), INTERFERin (Polyplus Transfection, New York, NY) and PULSin (Polyplus Transfection), respectively, were used according to the manufacturer's instructions. Cell culture reagents were purchased from Invitrogen. All other reagents were obtained from Sigma (Taufkirchen, Germany) if not stated otherwise.

### Bacterial strains and cell culture

*Salmonella enterica* subsp. *enterica* sv. Typhimurium (*S.* Typhimurium) ATCC 14028 was used as wildtype strain. Isogenic mutant strains (Δ*waaL* and Δ*waaG*) were generated by Red recombinase mediated deletion and chromosomal insertion of a Kanamicin antibiotic resistance cassette as described elsewhere (Zenk et al., submitted). The construction of plasmids for the complementation of mutant strains is described in (Zenk et al. submitted). The LPS profiles of the various strains were analyzed using SDS-PAGE and silver staining (Fig. S1A). Bacteria were incubated at 70°C for 10 min to produce heat-killed *Salmonella*. The non-invasive isogenic *pho-24* (PhoP constitutive) and Δ*hilA* mutants were a generous gift from Mikael Rhen (Karolinska Institute, Stockholm) and the isogenic Δ*invC* and Δ*invC* Δ*waaL* double mutants were generated as described above. HilA is a central regulator of SPI-I mediated epithelial cell invasion, the PhoP/PhoQ two component system is a central regulator in *Salmonella* virulence, and InvC is required for type III secretion of SPI-1-encoded virulence determinants. The phenotype of the non-invasive *pho-24* mutant is designated PhoP^constitutive^ (PhoP^c^). All three mutants exhibit strongly impaired epithelial invasion. Fluorescent bacteria were generated by transformation with a constitutively GFP expressing plasmid. For all experiments, bacteria were routinely grown in Luria-Bertani (LB) broth, supplemented with antibiotics if required. Murine small intestinal epithelial m-IC_cl2_ cells and m-IC_cl2_ cells stably expressing a NF-κB luciferase reporter construct were cultured as described previously [52]. RAW 264.7 macrophages were purchased from ATCC and cultured in RPMI 1640 medium (Invitrogen) supplemented with 20 mM Hepes, 2 mM L-glutamine, and 10% FCS.

### Bacterial coculture and stimulation assays

For all *coculture* experiments, wildtype or mutant *Salmonella* were grown overnight at 37°C, diluted 1∶10 and subcultivated with mild agitation at 37°C, until mid-logarithmic growth was reached (OD_600_: 0.5). Bacteria were adjusted by dilution, added to polarized and differentiated intestinal epithelial m-IC_cl2_ cells at a multiplicity of infection (MOI) of 10∶1 and centrifuged at 300×*g* for 5 min. Following incubation for one hour, the medium was replaced with fresh medium supplemented with 50 µg/mL Gentamicin. Cell culture supernatants, as well as cell lysates, were collected after the indicated periods of time and stored at −20°C. The chemokine MIP-2 was analyzed using a commercial ELISA from Nordic Biosite (Täby, Sweden). Luciferase in cell lysates was quantified using a luciferase reporter kit (Promega, Madison, WI). Pharmacological inhibitors were added to the cell medium 30 min prior to stimulation. NO production was determined by measurement of nitrite in cell culture supernatant using Griess reagent [53]. Stimulation with mouse recombinant TNF (R&D Systems GmbH, Wiesbaden, Germany) was performed at 100 ng/mL. To quantify bacterial invasion *coculture* for one hour was followed by one hour incubation in fresh cell culture medium, supplemented with 50 µg/mL Gentamicin. After washing, cells were lysed in H_2_O/Tween 0.1% and the number of intracellular bacteria was determined by serial dilution and plating. Specific or control siRNA was transfected with INTERFERin at a final concentration of 1 or 10 nM 48 hours prior to functional analysis. TLR4 blocking experiments using the rat monoclonal anti-TLR4/MD2 antibody MTS510 were conducted in the presence or absence of the protein delivery reagent PULSin according to the manufacturer's recommendations. R-Phycoerythrin (R-PE) is a fluorescent protein to visualize efficient protein transfection by PULSin (data not shown).

### Immunostaining and flow cytometric analysis

m-IC_cl2_ or RAW 264.7 cells were grown on 8-well chamber slides (Nunc, Rochester, NY), and infected with constitutively GFP-expressing wildtype *S.* Typhimurium or isogenic mutants as indicated at a MOI of 10∶1, or exposed to biotinylated LPS (100 ng/mL), and incubated for the specifically mentioned period of time. Visualization of the cellular distribution of p65/RelA was performed as previously described [15]. Discrimination of extra-and intracellular bacteria was achieved using *Salmonella* strains carrying a GFP expression construct (green) in combination with a mixture of two mouse monoclonal anti-*Salmonella* O-antigen (anti-O4 and anti-O5) antibodies visualized with a Texas-Red-conjugated anti-mouse secondary antibody (red) in the absence of cell permeabilization. Due to impaired penetration of the anti-*Salmonella* antibodies into the cells, intracellular bacteria appear green, whereas extracellular bacteria exhibit an orange (green plus red) color. Biotinylated LPS was detected using Texas-Red conjugated Streptavidin (Jackson ImmunoResearch). For permeabilization of eukaryotic cell membrane, saponin was adjusted to a final concentration of 0.5%. After the indicated time periods, cells were fixed in 5% PFA and counterstained with MFP488- or MFP647-phalloidin (MoBiTec, Göttingen, Germany). Cells were subsequently mounted in DAPI containing Vectashield (Vector Laboratories) and visualized using an ApoTome-equipped Axioplan 2 microscope connected to an AxioCam M_r_ digital Camera (Carl Zeiss MicroImaging, Inc. Jena, Germany). Flow cytometric detection of intracellular GFP-expressing bacteria in intact epithelial cells was carried out in Trypsin-EDTA 0.05% treated, fixed m-IC_cl2_ cells using a FACS Calibur® apparatus (BD Pharmingen). In addition, flow cytometry was used to quantify the number of GFP-expressing bacteria in cell lysates. To standardize the volume examined, a defined quantity of Cy5-labelled particles was added to all samples and the data acquisition on GFP-positive bacteria (recorded in channel Fl-1) was limited until a simultaneously recorded number of 10.000 events in the far red channel (Cy5, Fl-4) was reached.

### Bacterial challenge

Mice were purchased from Charles River Breeding Laboratories (Sulzfeld, Germany) and housed under specific pathogen-free conditions. 6–8-week-old female Balb/c mice were orally infected with 1×10^8^ CFU *S.* Typhimurium in 20 µl phosphate-buffered saline (PBS). 4 and 24 h post infection, mice were sacrificed and the small intestine was removed. Highly pure IECs (>98% E-cadherin^+^/CD45^−^) were isolated using a recently described protocol [15], incubated in the presence of Gentamicin (50 µg/mL) and washed. The number of viable intraepithelial bacteria was determined by serial plating.

### Statistical analysis

All experiments were performed at least three times and results are given as the mean±SD of one representative experiment. Statistical analyses were performed using the Student's *t* test. A p value<0.05 was considered significant.

## Supporting Information

Figure S1 (A) Characterization of the LPS produced by the wildtype, *waaL* and *waaG* mutant, as well as the respective complemented *waaG* [*waaG*] and *waaL* [*waaL*] *Salmonella* strains used in this study. LPS extracts from equal numbers of bacterial cells (3×10^9^ CFU) were loaded in each lane and analyzed by SDS-PAGE using a 12% acrylamide gel subsequently followed by silver staining. The relative positions of the lipid A, inner core, outer core, and O-antigen are indicated. (B) Kinetic of MIP2 secretion by m-IC_cl2_ cells in response to exposure to heat-killed wildtype and *waaL* mutant O-antigen-deficient *Salmonella*. (C and D) Quantitative flow cytometric analysis of intracellular GFP expressing wildtype and *waaL*-deficient *Salmonella* 30 min. after infection. (C) Dot blot analysis and (D) normalized numbers of GFP-positive bacteria detected in a defined volume of epithelial cell lysate 30 min. after infection. (E) Growth of wildtype (wt), *waaG* and *waaL*-mutant, as well as the respective complemented strains *waaG* [*waaG*] and *waaL* [*waaL*] in m-IC_cl2_ cell lysate diluted 1∶3 in phosphate buffered saline (PBS). The number of colony forming units (CFU) normalized to the inoculum is indicated at each time point. (F) Viable intracellular wildtype or *waaL*-deficient *Salmonella* 2, 4, and 6 hours after infection of naive m-IC_cl2_ cells. Gentamicin (50 µg/mL) was added to the cell culture medium one hour after addition of the bacteria. (G, H, and I) Analysis of the purity of the LPS preparation used in this study. NO release (G) and MIP2 secretion (H) by LPS-stimulated [100 ng/mL] wildtype or *Tlr4*-deficient peritoneal macrophages. (I) *Tlr4*-deficient peritoneal macrophages readily responded to the proinflammatory cytokine TNF but not LPS [10 ng/mL]. CFU, colony forming units; n.d., not detectable. **, p<0.01.
(0.45 MB TIF)

Figure S2 (A) The non-invasive phenotype of the *S.* Typhimurium *pho-24* (PhoP^c^) and *hilA* mutants [MOI 10∶1] demonstrated by a Gentamicin protection-assay. (B) Stably transfected m-IC_cl2_ cells expressing a NF-κB-luciferase construct were co-incubated with wildtype *S.* Typhimurium, or isogenic non-invasive *pho-24* (PhoP^c^) and *hilA* mutants [MOI 10∶1] for 2 hours, or 6 hours, and the amount of luciferase was quantified. **, p<0.01. (C) Flow cytometric analysis of RAW 264.7 cells left untreated or 30 and 120 min after infection with GFP-expressing wildtype or *waaL*-deficient *Salmonella*. The left lower panel illustrates the number of *Salmonella*-positive RAW 264.7 macrophages [%] at 30 and 120 min after infection. Note the significantly enhanced invasion rate of rough as compared to smooth *Salmonella* in macrophages. (D) Viable intracellular wildtype *Salmonella* two hours after infection of m-IC_cl2_ cells [MOI 10∶1] as measured by Gentamicin protection-assay. Cells were pretreated with smooth LPS or rough LPS [100 ng/mL] for 20 min prior to infection. *, p<0.05. (E) Susceptibility against serum bactericidial activity of the *Salmonella* wildtype, *waaL* mutant, and complemented strain. 10^3^ *Salmonella* were incubated in 20% fresh human serum or inactivated serum (56°C for 30 min) for 0, 15, and 30 min and the number of viable bacteria was determined by serial dilution plating. n.d., not detectable; **, p<0.01.
(0.48 MB TIF)

## References

[1] Raetz CR, Whitfield C (2002). Lipopolysaccharide endotoxins.. Annu Rev Biochem.

[2] Montminy SW, Khan N, McGrath S, Walkowicz MJ, Sharp F (2006). Virulence factors of *Yersinia pestis* are overcome by a strong lipopolysaccharide response.. Nat Immunol.

[3] Poltorak A, He X, Smirnova I, Liu MY, Van Huffel C (1998). Defective LPS signaling in C3H/HeJ and C57BL/10ScCr mice: mutations in Tlr4 gene.. Science.

[4] Murray GL, Attridge SR, Morona R (2006). Altering the length of the lipopolysaccharide O antigen has an impact on the interaction of *Salmonella ente*rica serovar Typhimurium with macrophages and complement.. J Bacteriol.

[5] Gioannini TL, Weiss JP (2007). Regulation of interactions of Gram-negative bacterial endotoxins with mammalian cells.. Immunol Res.

[6] Jerala R (2007). Structural biology of the LPS recognition.. Int J Med Microbiol.

[7] Park BS, Song DH, Kim HM, Choi BS, Lee H (2009). The structural basis of lipopolysaccharide recognition by the TLR4-MD-2 complex.. Nature.

[8] Schilling JD, Martin SM, Hung CS, Lorenz RG, Hultgren SJ (2003). Toll-like receptor 4 on stromal and hematopoietic cells mediates innate resistance to uropathogenic *Escherichia coli*.. Proc Natl Acad Sci USA.

[9] Andonegui G, Bonder CS, Green F, Mullaly SC, Zbytnuik L (2003). Endothelium-derived Toll-like receptor-4 is the key molecule in LPS-induced neutrophil sequestration into lungs.. J Clin Invest.

[10] Weiss DS, Raupach B, Takeda K, Akira S, Zychlinsky A (2004). Toll-like receptors are temporally involved in host defense.. J Immunol.

[11] Rakoff-Nahoum S, Paglino J, Eslami-Varzaneh F, Edberg S, Medzhitov R (2004). Recognition of commensal microflora by toll-like receptors is required for intestinal homeostasis.. Cell.

[12] Rakoff-Nahoum S, Medzhitov R (2007). Regulation of spontaneous intestinal tumorigenesis through the adaptor protein MyD88.. Science.

[13] Hornef MW, Frisan T, Vandewalle A, Normark S, Richter-Dahlfors A (2002). Toll-like receptor 4 resides in the Golgi apparatus and colocalizes with internalized lipopolysaccharide in intestinal epithelial cells.. J Exp Med.

[14] Latz E, Visintin A, Lien E, Fitzgerald KA, Monks BG (2002). Lipopolysaccharide rapidly traffics to and from the Golgi apparatus with the toll-like receptor 4-MD-2-CD14 complex in a process that is distinct from the initiation of signal transduction.. J Biol Chem.

[15] Lotz M, Gütle D, Walther S, Ménard S, Bogdan C (2006). Postnatal acquisition of endotoxin tolerance in intestinal epithelial cells.. J Exp Med.

[16] Hornef MW, Normark BH, Vandewalle A, Normark S (2003). Intracellular recognition of lipopolysaccharide by toll-like receptor 4 in intestinal epithelial cells.. J Exp Med.

[17] Dunzendorfer S, Lee HK, Soldau K, Tobias PS (2004). Toll-like receptor 4 functions intracellularly in human coronary artery endothelial cells: roles of LBP and sCD14 in mediating LPS responses.. FASEB J.

[18] Thieblemont N, Thieringer R, Wright SD (1998). Innate immune recognition of bacterial lipopolysaccharide: dependence on interactions with membrane lipids and endocytic movement.. Immunity.

[19] Ueta M, Nochi T, Jang MH, Park EJ, Igarashi O (2004). Intracellularly expressed TLR2s and TLR4s contribution to an immunosilent environment at the ocular mucosal epithelium.. J Immunol.

[20] Guillot L, Medjane S, Le-Barillec K, Balloy V, Danel C (2004). Response of human pulmonary epithelial cells to lipopolysaccharide involves Toll-like receptor 4 (TLR4)-dependent signaling pathways: evidence for an intracellular compartmentalization of TLR4.. J Biol Chem.

[21] Dunzendorfer S, Lee HK, Soldau K, Tobias PS (2004). TLR4 is the signaling but not the lipopolysaccharide uptake receptor.. J Immunol.

[22] Chassin C, Hornef MW, Bens M, Lotz M, Goujon JM (2007). Hormonal control of the renal immune response and antibacterial host defense by arginine vasopressin.. J Exp Med.

[23] Creeger ES, Rothfield LI (1979). Cloning of genes for bacterial glycosyltransferases. I. Selection of hybrid plasmids carrying genes for two glucosyltransferases.. J Biol Chem.

[24] Heinrichs DE, Yethon JA, Whitfield C (1998). Molecular basis for structural diversity in the core regions of the lipopolysaccharides of *Escherichia coli* and *Salmonella enterica*.. Mol Microbiol.

[25] Hamann L, Alexander C, Stamme C, Zähringer U, Schumann RR (2005). Acute-phase concentrations of lipopolysaccharide (LPS)-binding protein inhibit innate immune cell activation by different LPS chemotypes via different mechanisms.. Infect Immun.

[26] Ilg K, Endt K, Misselwitz B, Stecher B, Aebi M (2009). O-antigen-negative *Salmonella enterica* serovar Typhimurium is attenuated in intestinal colonization but elicits colitis in streptomycin-treated mice.. Infect Immun.

[27] Takeuchi A (1967). Electron microscope studies of experimental *Salmonella* infection. I. Penetration into the intestinal epithelium by *Salmonella* typhimurium.. Am J Pathol.

[28] Ellermeier JR, Slauch JM (2007). Adaptation to the host environment: regulation of the SPI1 type III secretion system in *Salmonella enterica* serovar Typhimurium.. Curr Opin Microbiol.

[29] Frost AJ, Bland AP, Wallis TS (1997). The early dynamic response of the calf ileal epithelium to *Salmonella* typhimurium.. Vet Pathol.

[30] Hapfelmeier S, Stecher B, Barthel M, Kremer M, Müller AJ (2005). The *Salmonella* pathogenicity island (SPI)-2 and SPI-1 type III secretion systems allow *Salmonella* serovar typhimurium to trigger colitis via MyD88-dependent and MyD88-independent mechanisms.. J Immunol.

[31] Kim CH (2003). A Salmonella typhimurium rfaE mutant recovers invasiveness for human epithelial cells when complemented by wild type rfaE (controlling biosynthesis of ADP-L-glycero-D-mannoheptose-containing lipopolysaccharide).. Mol Cells.

[32] Bruggen van R, Zweers D, Diepen van A, Dissel van JT, Roos D (2007). Complement receptor 3 and Toll-like receptor 4 act sequentially in uptake and intracellular killing of unopsonized *Salmonella enterica* serovar Typhimurium by human neutrophils.. Infect Immun.

[33] Roy MF, Larivière L, Wilkinson R, Tam M, Stevenson MM (2006). Incremental expression of *Tlr4* correlates with mouse resistance to *Salmonella* infection and fine regulation of relevant immune genes.. Genes Immun.

[34] Leveque G, Forgetta V, Morroll S, Smith AL, Bumstead N (2003). Allelic variation in TLR4 is linked to susceptibility to *Salmonella enterica* serovar Typhimurium infection in chickens.. Infect Immun.

[35] Bernheiden M, Heinrich JM, Minigo G, Schütt C, Stelter F (2001). LBP, CD14, TLR4 and the murine innate immune response to a peritoneal *Salmonella* infection.. J Endotoxin Res.

[36] Miller SI, Ernst RK, Bader MW (2005). LPS, TLR4 and infectious disease diversity.. Nat Rev.Microbiol.

[37] Huber M, Kalis C, Keck S, Jiang Z, Georgel P (2006). R-form LPS, the master key to the activation ofTLR4/MD-2-positive cells.. Eur J Immunol.

[38] Jiang Z, Georgel P, Du X, Shamel L, Sovath S (2005). CD14 is required for MyD88-independent LPS signaling.. Nat Immunol.

[39] Samuel G, Reeves P (2003). Biosynthesis of O-antigens: genes and pathways involved in nucleotide sugar precursor synthesis and O-antigen assembly.. Carbohydr Res.

[40] Rautemaa R, Meri S (1999). Complement-resistance mechanisms of bacteria.. Microbes Infect.

[41] Guo L, Lim KB, Gunn JS, Bainbridge B, Darveau RP (1997). Regulation of lipid A modifications by Salmonella typhimurium virulence genes phoP-phoQ.. Science.

[42] Kastowsky M, Gutberlet T, Bradaczek H (1992). Molecular modelling of the three-dimensional structure and conformational flexibility of bacterial lipopolysaccharide.. J Bacteriol.

[43] Beveridge TJ (1999). Structures of gram-negative cell walls and their derived membrane vesicles.. J Bacteriol.

[44] Lam JS, Graham LL, Lightfoot J, Dasgupta T, Beveridge TJ (1992). Ultrastructural examination of the lipopolysaccharides of *Pseudomonas aeruginosa* strains and their isogenic rough mutants by freeze-substitution.. J Bacteriol.

[45] West NP, Sansonetti P, Mounier J, Exley RM, Parsot C (2005). Optimization of virulence functions through glucosylation of *Shigella* LPS.. Science.

[46] Steele-Mortimer O, Brumell JH, Knodler LA, Méresse S, Lopez A (2002). The invasion-associated type III secretion system of *Salmonella enterica* serovar Typhimurium is necessary for intracellular proliferation and vacuole biogenesis in epithelial cells.. Cell Microbiol.

[47] Holden DW (2002). Trafficking of the *Salmonella* vacuole in macrophages.. Traffic.

[48] Blander JM, Medzhitov R (2004). Regulation of phagosome maturation by signals from toll-like receptors.. Science.

[49] Rittig MG, Kaufmann A, Robins A, Shaw B, Sprenger H (2003). Smooth and rough lipopolysaccharide phenotypes of *Brucella* induce different intracellular trafficking and cytokine/chemokine release in human monocytes.. J Leukoc Biol.

[50] Nnalue NA, Lindberg AA (1990). *Salmonella choleraesuis* strains deficient in O antigen remain fully virulent for mice by parenteral inoculation but are avirulent by oral administration.. Infect Immun.

[51] Bogomolnaya LM, Santiviago CA, Yang HJ, Baumler AJ, Andrews-Polymenis HL (2008). ‘Form variation’ of the O12 antigen is critical for persistence of *Salmonella* Typhimurium in the murine intestine.. Mol Microbiol.

[52] Bens M, Bogdanova A, Cluzeaud F, Miquerol L, Kerneis S (1996). Transimmortalized mouse intestinal cells (m-ICc12) that maintain a crypt phenotype.. Am J Physiol.

[53] Colasanti M, Persichini T, Menegazzi M, Mariotto S, Giordano E (1995). Induction of nitric oxide synthase mRNA expression. Suppression by exogenous nitric oxide.. J Biol Chem.

