# Supplementary figures and images for: O-Antigen Delays Lipopolysaccharide Recognition and Impairs Antibacterial Host Defense in Murine Intestinal Epithelial Cells
Source: PLoS Pathog. 2009 Sep 4;5(9):e1000567. doi: 10.1371/journal.ppat.1000567 (PMC2729928; doi:10.1371/journal.ppat.1000567)

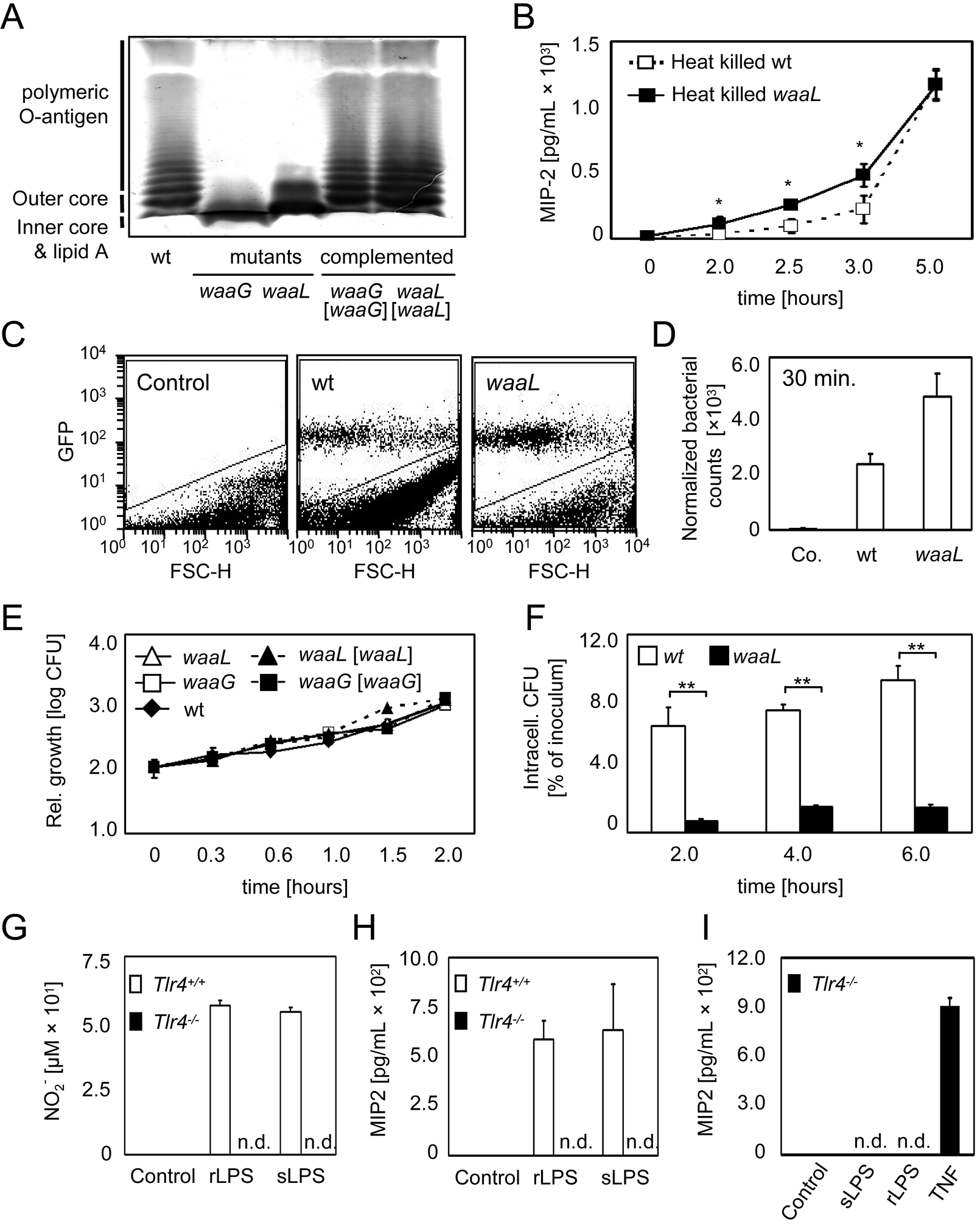

Supplement: Figure S1 — (A) Characterization of the LPS produced by the wildtype, waaL and waaG mutant, as well as the respective complemented waaG [waaG] and waaL [waaL] Salmonella strains used in this study. LPS extracts from equal numbers of bacterial cells (3×10^9^ CFU) were loaded in each lane and analyzed by SDS-PAGE using a 12% acrylamide gel subsequently followed by silver staining. The relative positions of the lipid A, inner core, outer core, and O-antigen are indicated. (B) Kinetic of MIP2 secretion by m-ICcl2 cells in response to exposure to heat-killed wildtype and waaL mutant O-antigen-deficient Salmonella. (C and D) Quantitative flow cytometric analysis of intracellular GFP expressing wildtype and waaL-deficient Salmonella 30 min. after infection. (C) Dot blot analysis and (D) normalized numbers of GFP-positive bacteria detected in a defined volume of epithelial cell lysate 30 min. after infection. (E) Growth of wildtype (wt), waaG and waaL-mutant, as well as the respective complemented strains waaG [waaG] and waaL [waaL] in m-ICcl2 cell lysate diluted 1∶3 in phosphate buffered saline (PBS). The number of colony forming units (CFU) normalized to the inoculum is indicated at each time point. (F) Viable intracellular wildtype or waaL-deficient Salmonella 2, 4, and 6 hours after infection of naive m-ICcl2 cells. Gentamicin (50 µg/mL) was added to the cell culture medium one hour after addition of the bacteria. (G, H, and I) Analysis of the purity of the LPS preparation used in this study. NO release (G) and MIP2 secretion (H) by LPS-stimulated [100 ng/mL] wildtype or Tlr4-deficient peritoneal macrophages. (I) Tlr4-deficient peritoneal macrophages readily responded to the proinflammatory cytokine TNF but not LPS [10 ng/mL]. CFU, colony forming units; n.d., not detectable. **, p<0.01. (0.45 MB TIF) [file ppat.1000567.s001.tif]

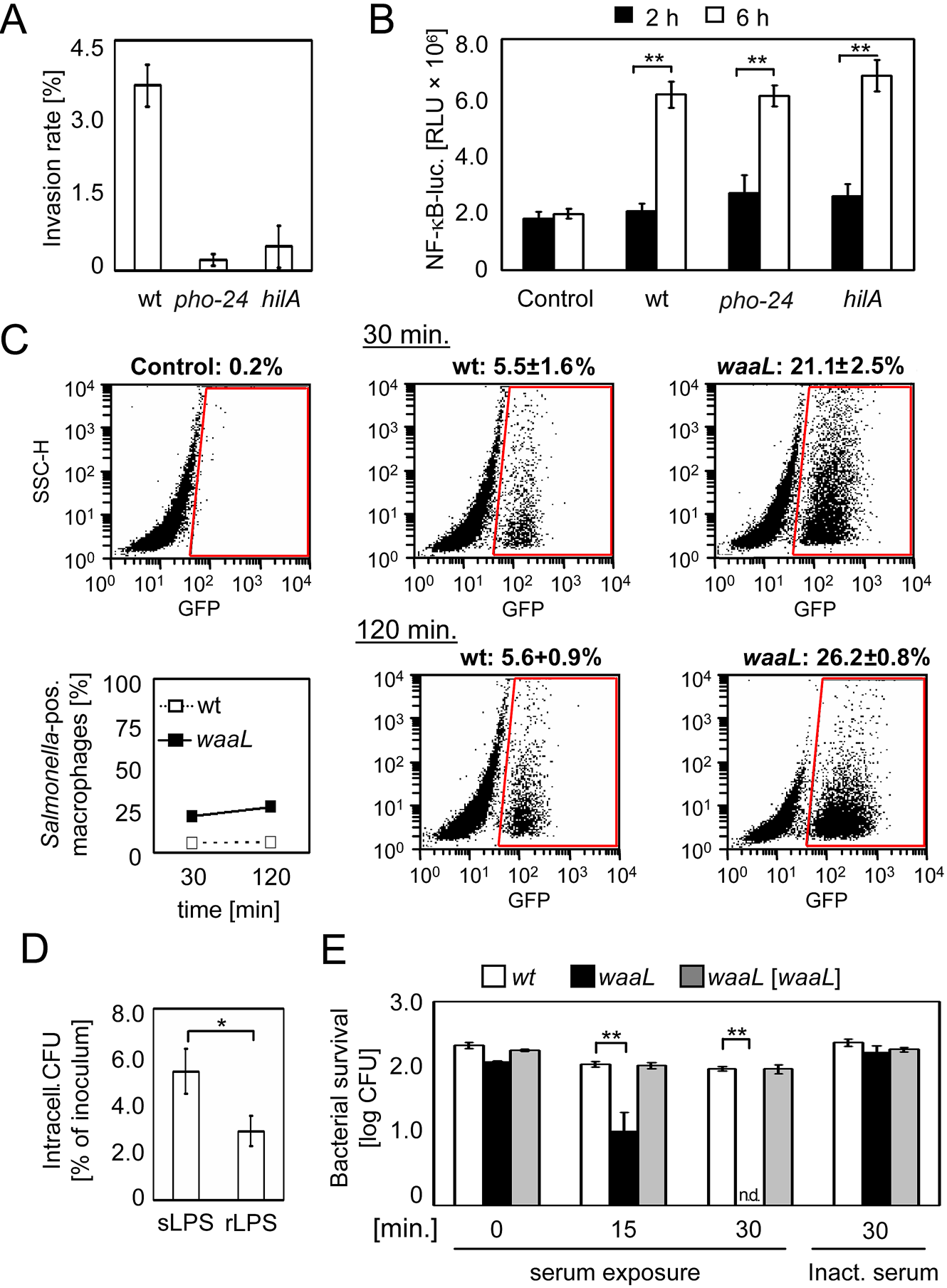

Supplement: Figure S2 — (A) The non-invasive phenotype of the S. Typhimurium pho-24 (PhoPc) and hilA mutants [MOI 10∶1] demonstrated by a Gentamicin protection-assay. (B) Stably transfected m-ICcl2 cells expressing a NF-κB-luciferase construct were co-incubated with wildtype S. Typhimurium, or isogenic non-invasive pho-24 (PhoPc) and hilA mutants [MOI 10∶1] for 2 hours, or 6 hours, and the amount of luciferase was quantified. **, p<0.01. (C) Flow cytometric analysis of RAW 264.7 cells left untreated or 30 and 120 min after infection with GFP-expressing wildtype or waaL-deficient Salmonella. The left lower panel illustrates the number of Salmonella-positive RAW 264.7 macrophages [%] at 30 and 120 min after infection. Note the significantly enhanced invasion rate of rough as compared to smooth Salmonella in macrophages. (D) Viable intracellular wildtype Salmonella two hours after infection of m-ICcl2 cells [MOI 10∶1] as measured by Gentamicin protection-assay. Cells were pretreated with smooth LPS or rough LPS [100 ng/mL] for 20 min prior to infection. *, p<0.05. (E) Susceptibility against serum bactericidial activity of the Salmonella wildtype, waaL mutant, and complemented strain. 103 Salmonella were incubated in 20% fresh human serum or inactivated serum (56°C for 30 min) for 0, 15, and 30 min and the number of viable bacteria was determined by serial dilution plating. n.d., not detectable; **, p<0.01. (0.48 MB TIF) [file ppat.1000567.s002.tif]
